# The Significance of Reactive Oxygen Species and Antioxidant Defense System in Plants: A Concise Overview

**DOI:** 10.3389/fpls.2020.552969

**Published:** 2021-01-06

**Authors:** Jelena Dumanović, Eugenie Nepovimova, Maja Natić, Kamil Kuča, Vesna Jaćević

**Affiliations:** ^1^Faculty of Chemistry, University of Belgrade, Belgrade, Serbia; ^2^Medical Faculty of the Military Medical Academy, University of Defence, Belgrade, Serbia; ^3^Department of Chemistry, Faculty of Science, University of Hradec Kralove, Hradec Králové, Czechia; ^4^Department for Experimental Toxicology and Pharmacology, National Poison Control Centre, Military Medical Academy, Belgrade, Serbia

**Keywords:** oxidative stress, reactive oxygen species, antioxidative defence system, cell, plants

## Abstract

In plants, there is a complex and multilevel network of the antioxidative system (AOS) operating to counteract harmful reactive species (RS), the foremost important of which are reactive oxygen species (ROS), and maintain homeostasis within the cell. Specific AOSs for plant cells are, first and foremost, enzymes of the glutathione-ascorbate cycle (Asc-GSH), followed by phenolic compounds and lipophilic antioxidants like carotenoids and tocopherols. Evidence that plant cells have excellent antioxidative defense systems is their ability to survive at H_2_O_2_ concentrations incompatible with animal cell life. For the survival of stressed plants, it is of particular importance that AOS cooperate and participate in redox reactions, therefore, providing better protection and regeneration of the active reduced forms. Considering that plants abound in antioxidant compounds, and humans are not predisposed to synthesize the majority of them, new fields of research have emerged. Antioxidant potential of plant compounds has been exploited for anti-aging formulations preparation, food fortification and preservation but also in designing new therapies for diseases with oxidative stress implicated in etiology.

## Introduction

Plants are multicellular organisms which, thanks to their inability to makeover, have very well-developed adaptation systems and mechanisms of protection to varying environmental conditions. External factors like drought, high and low temperatures; also as high levels of radiation have an adverse effect on plants. A standard characteristic of varied stressors is their potential to promote the generation of reactive oxygen species (ROS) in plant tissue, the build-up of which within the cell causes oxidative stress. This term was initially introduced by Sies and Cadenas (1985). Namely, oxidative stress implies an interruption of the redox equilibrium as a consequence of the increased level of ROS within the cell itself; however, the most recent version of definition may be “imbalance between oxidants and antioxidants in favor of the oxidants, leading to a disruption of redox signaling and control and/or molecular damage” (Sies, 2018). Also, recently, oxidative stress is classified in subforms: oxidative stress present in physiological conditions (eustress), and oxidative stress expressing deleterious effects on macromolecules (distress; Lushchak, 2014).

Paradoxically, oxygen as a molecule which sustains aerobic life, against being essential for energy metabolism and respiration, is involved within the mechanism of the onset of various diseases and degenerative conditions (Sies et al., 2017). With the evolution of photosynthesis, initially by cyanobacteria and afterwards by plants, over 2 billion years ago, the quantity of oxygen on Earth has increased significantly. Molecular oxygen is made as a by-product during this process by operation of the oxygen-evolving complex (OEC), which is a component of the photosystem (PS) II (Yachandra et al., 1996). The massive quantities of present oxygen enabled the production of more ATP *via* aerobic respiration but also increased the danger of ROS formation. Aerobic organisms are ready to survive by virtue of the event of antioxidant protection mechanisms, exhibiting a defensive role against a vast number of ROS (Dumont and Rivoal, 2019).

Molecular oxygen can act as an oxidant, but despite its high thermodynamic reactivity, its reactions are kinetically slow thanks to the prevailing spin restriction (Krieger-Liszkay, 2005). In its ground state, oxygen appears as a triplet (^3^O_2_), with two unpaired electrons (biradical) of parallel spins in two separate orbitals, which makes it paramagnetic and thus shows no affinity for organic molecules unless activated. Oxygen activation is often achieved by two mechanisms (Apel and Hirt, 2004):

1. Absorbing excess energy sufficient to rotate the spin of one unpaired electron to make a single state (^1^O_2_), during which two electrons are of opposite spin.
2. A multi-step monovalent reduction to the formation of superoxide radical (O_2_^•−^), hydrogen peroxide (H_2_O_2_), hydroxyl radical (^•^OH), and eventually water.

By activation, the spin restriction has been surpassed and ^1^O_2_ can interact in two-electron transfer reactions, while its oxidizing capacity is greatly increased. The gradual reduction of triplet oxygen, exposing to high energy or electron transfer reactions, results in the formation of ROS (Sharma et al., 2012). Numerous defense mechanisms are implicated within the battle against these highly reactive molecules, the foremost important being the antioxidative system (AOS). The goal of such a system is to guard cells against ROS and oxidative stress that happens if the influence of ROS prevails (Huang et al., 2019). When determining if some molecule would behave as anti‐ or prooxidant of particular importance are micro-conditions (pH, presence of trace metals, etc.) restricted for specific cell compartment.

In this short review paper, we have selected the ROS, briefly summarized their main characteristics, described their prooxidant activities, and outlined the most prominent antioxidants in plants.

## Reactive Oxygen Species

Reactive species (RS) are a broad term and include ROS, nitrogen [reactive nitrogen species (RNS)], sulfur [reactive sulfur species (RSS)], and other species, several of which are free radicals, and each has the potential to cause oxidative stress as a result of their accumulation within the cell to a level that exceeds the capacity to remove them (Mittler, 2017). ROS are the most vital group of RS and include, additionally to free radicals, non-radical forms which do not have unpaired electrons but also are highly reactive, e.g., H_2_O_2_, ^1^O_2_, hypochlorous acid (HClO), and ozone (O_3_). Free radicals are known since the twentieth century within the world of chemistry and were originally described as intermediate compounds in organic and inorganic chemistry (Kohen and Nyska, 2002). These molecules that have one or more unpaired electrons, leading to high reactivity, are formed when an atom or molecule “loses” or “gains” one electron, or during homolytic cleavage of a covalent bond. Conversely, when two free radicals share their unpaired electrons, non-radical species are formed (Arora et al., 2002). The term “Reactive” may be a relative term, while ^•^OH indiscriminately reacts with all biological molecules in its vicinity, O_2_^•−^ and H_2_O_2_ are highly selective. From the pathophysiological, as well as the physiological, point of view, the foremost significant ROS are ^•^OH, O_2_^•−^, organic alkoxy (RO^•^), and organic peroxyl radicals (ROO^•^) as well as non-radical species: ^1^O_2_, H_2_O_2_, and O_3_ (Dumont and Rivoal, 2019).

**Figure 1:**
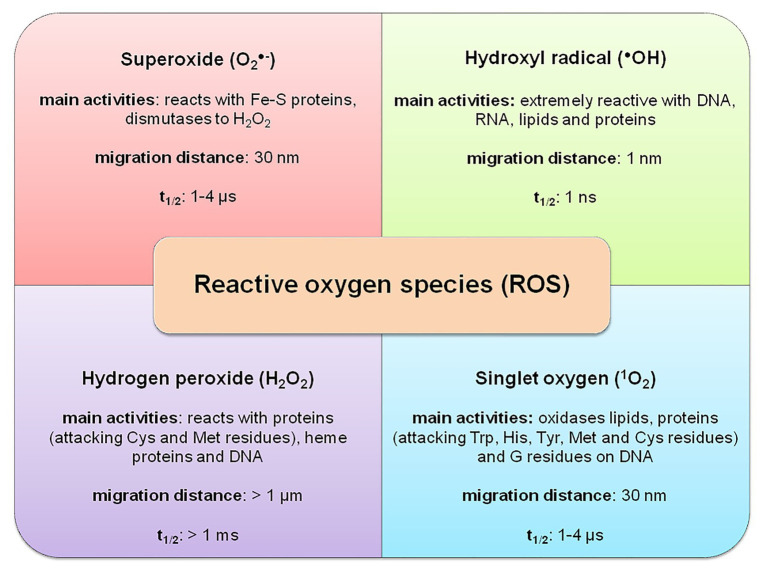
Basic properties of reactive oxygen species (ROS) in a plant cell – adapted from: Mittler (2017; https://creativecommons.org/licenses/by-nc-nd/4.0/).

Toxicity is not necessarily associated with reactivity. In many cases, the longer half-life of ROS provides an extended time for diffusion and consequently the power to succeed in sensitive sites within a cell where it can react with biomolecules far away from the location of its generation. For instance, the relatively long-lived O_2_^•−^ (with a half-life of 1–4 μs and migration distance of 30 nm) generated on the mitochondrial membrane, diffuses toward the mitochondrial genome and reduces the transition metals within the genome itself. Singlet oxygen has approximately equivalent properties as O_2_^•−^ with the best affinity to Trp, His, Tyr, and Cys residues of proteins. Hydrogen peroxide could live quite 1 ms, and its migration distance is in range of 1 μm, enabling to react with DNA and Cys and Met residues of protein far away from its origin (Mittler, 2017). On the contrary, extremely reactive ^•^OH features a half-life of roughly 1 ns and migration distance of 1 nm, therefore, reacting with all neighboring biomolecules like DNA, RNA, lipids, and proteins (Figure 1).

To prevent the interaction between radicals and biological molecules, antioxidants should be in close vicinity to the radical’s place of formation, being in competition with the free radical for the biological substrate (Arora et al., 2002). If an antioxidant is not present in sufficient quantity to neutralize ROS, oxidation of biomolecules, like lipid peroxidation, protein damage (carbonylation of aminoalkanoic acid residues), oxidation of single DNA and RNA nucleotides, enzyme inhibition, and activation of apoptosis, will occur (Gill and Tuteja, 2010).

One of the foremost studied ROS is hydrogen peroxide. It exhibits a dual role: in low concentrations, it participates in signal transduction, while in high concentrations, it exerts a toxic effect on the cell. Under physiological conditions, the extent of H_2_O_2_ in leaves ranges approximately 1 μmol per gram of fresh tissue weight, i.e., 10 μmol/L H_2_O_2_ in peroxisomes (Cheeseman, 2006; Foyer and Noctor, 2016). Its presence in apoplast is essential for normal cell development. Acting as a substrate for class III peroxidase, H_2_O_2_ participates in phenolic compounds’ oxidation and, consequently, polymerization and cell wall lignin formation (Lewis and Yamamoto, 1990; Almagro et al., 2008; Moural et al., 2017; Pandey et al., 2017). On the other side, H_2_O_2_ expresses toxicity for several cells within the concentration range of 10–100 μmol/L, leading to aging or apoptosis. The mechanism of its toxicity is direct inactivation of enzymes by oxidation of cysteine (–SH) or methionine (–SCH_3_) residues necessary for catalysis. An indirect effect of H_2_O_2_ is expressed by crossing the cellular membranes *via* peroxiporins and by reacting with the Fe^2+^ or Cu^+^ ions, resulting in the formation of more potent toxic species such as ^•^OH, and exactly ^•^OH is liable for the bulk, or maybe all of the damage caused to DNA molecules in cells treated with H_2_O_2_. Sometimes exposure of cells to H_2_O_2_ could increase O_2_^•−^ production due to NOX (EC 1.6.3.1) activation. Furthermore, H_2_O_2_ has long been recognized as a potent inhibitor of photosynthesis since, even at low H_2_O_2_ concentrations (10 μmol/L) could inhibit CO_2_ fixation by 50% by oxidizing enzymes included in Calvin cycle (Foyer and Shigeoka, 2011).

Plants are especially exposed to oxidative stress caused by ^1^O_2_ since they are rich in chlorophyll (Chl) which acts as a photosensitizer, and ^1^O_2_ is consistently generated in leaves. Chlorophyll is an efficient pigment which absorbs light within the so-called light-harvesting complexes (LHCs), intrinsic antennas, and PS II reaction centers, with the extra advantage that its excited state is long-lived enough to supply excitatory energy conversion to electrochemical potential *via* the method of charge separation during photosynthesis (Krieger-Liszkay, 2005; Xiulan et al., 2019). However, the excited (triplet) state of chlorophyll (^3^Chl) could supply nearby molecular oxygen with sufficient energy, leading to ^1^O_2_ formation if the energy is not efficiently used, or effective scavenger is lacking. Singlet oxygen is taken into account to be the foremost important ROS liable for the light-induced loss of PS II activity *via* degradation of the D1 protein, also as for the so-called “bleaching” of pigment (Krieger-Liszkay, 2005). Plants use two strategies to guard the photosynthetic apparatus from photoinhibition. The primary is non-photochemical quenching, i.e., dissipation of excessive excitation energy of ^3^Chl in antennas of PS II within the sort of heat. Second, carotenoid-dependent mechanism, or “*quenching*,” is predicated on the power of PS II to transfer electrons to varied acceptors in its proximity (carotenoids, α-tocopherol), which liberate excess energy within the sort of heat, returning to their ground state (Trebst, 2003; Apel and Hirt, 2004). Although this chlorophyll-carotenoid transfer is extremely effective, still 5% of ^3^Chl remains and this incomplete quenching is the evidence that antenna pigments are considered as a possible source of ^1^O_2_ in chloroplasts with a tendency for damaging D1 protein (Triantaphylidès and Havaux, 2009).

Superoxide anion radical (O_2_^•−^) is the primary cell-generated ROS which triggers a cascade of reactions and, therefore, the formation of secondary ROS, either directly or *via* enzymatic and metal-catalyzed processes, counting on the cell compartment. Generated *via* single-electron reduction of molecular oxygen within the cell, it is rapidly converted to H_2_O_2_ by superoxide dismutase (SOD, EC 1.15.1.1) activity, preventing the build-up of O_2_^•−^, and thereupon damage and inactivation of proteins containing Fe-S clusters (Schieber and Chandel, 2014). The foremost important reaction of O_2_^•−^ is dismutation, non-enzymatic, or by SOD, where it reacts with another molecule of O_2_^•−^ resulting in oxidation of its one molecule to oxygen and reduction of the second to H_2_O_2_. The reaction is the most effective in acidic pH, and on the contrary, slower in the basic environment (Kohen and Nyska, 2002; Birben et al., 2012; Noctor et al., 2018).

Hydroxyl radical (^•^OH) is taken into account as the most potent oxidant which, owing to its short half-life, very positive redox potential (close to +2 V) and high affinity for biomolecules, non-selectively oxidize DNA, proteins, lipids, amino acids, sugars, and metals, leading to damage or genetic instability. Generally, ^•^OH is formed from H_2_O_2_ within the presence of iron or copper ions within the reaction described as well-known Fenton reaction (Fenton, 1984). For this reason, cells have various mechanisms for maintaining iron homeostasis, since no enzymes are found within the cell to eliminate ^•^OH (Schieber and Chandel, 2014). As a consequence of metal absorption from the soil, plants are more suspectable to oxidative stress, and special care is taken in preventing reaction between transition metals and H_2_O_2_ by their sequestration. For this reason, plants are heeled with ferritins and metallothioneins capable of storing iron, copper, and zinc (Halliwell and Gutteridge, 2015). Prooxidants, such as O_2_^•−^ and Asc, could re-reduce Fe^3+^ (Cu^2+^) to Fe^2+^ (Cu^1+^) and restore these ions in the game (Salehi et al., 2020).

### Sources of ROS in Plant Cells

**Figure 2:**
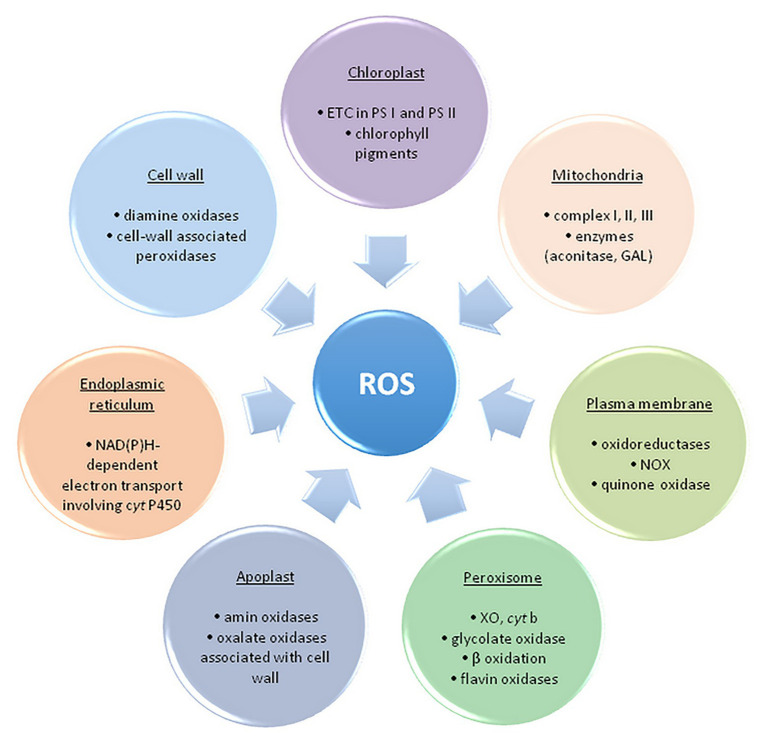
The main sites of ROS formation in a plant cell. ETC, electron transport chain; PS, photosystem; NOX, NAD(P)H oxidase; GAL, galactono-γ lactone dehydrogenase; XO, xanthine oxidase – adapted from: Sharma et al. (2012; https://creativecommons.org/licenses/by/3.0/).

Free radicals and other oxygen derivatives are inevitable by-products of biological redox reactions, as well as a consequence of aerobic metabolism in plants (Figure 2).

ROS are primarily formed in chloroplasts, mitochondria, plasma membranes, peroxisomes, apoplast, and endoplasmic reticulum (Elstner, 1982; Sharma et al., 2012). The maximum amount as 1% of the oxygen employed by plants is diverted to ROS synthesis in several cell compartments (Bhattacharjee, 2005). The main source of ROS in plants is photosynthesis, precisely, electron transport chain (ETC) and photorespiration in peroxisomes (Foyer and Shigeoka, 2011). Photosystems within ETC, PS II and PS I, respectively, generate ^1^O_2_ and O_2_^•−^ within the so-called Mehler reaction (Mehler and Brown, 1956; Apel and Hirt, 2004).

Additionally, production of ROS (primarily O_2_^•−^ and H_2_O_2_) occurs when molecular oxygen is reduced by mainly electron leakage in mitochondrial complex I and III (about 1–5% of oxygen is converted to H_2_O_2_; Rhoads et al., 2006; Černý et al., 2018). In peroxisomes, the process of photorespiration (glycolate pathway) takes place in C3 plants, during which glycolate formed within the chloroplast stroma is oxidized. Hydrogen peroxide is made as a by-product, and peroxisomes are considered the main site of its intracellular production. Homologues of NAD(P)H oxidase (NOX, EC 1.6.3.1) are found in numerous plants and are named the respiratory burst oxidase homolog (RBOH). RBOHs catalyze the transfer of electrons from NAD(P)H within the cytoplasm to the molecular oxygen and create O_2_^•−^ during the defense of plants against pathogens (Bhattacharjee, 2005). Additionally, to the cell wall, RBOHs are also expressed in vacuoles, endoplasmic reticulum, nucleus, and mitochondria (Mittler, 2017). Oxidative burst is taken into account one among the plant’s main responses to (a)biotic stress (high intensity of UV and photosynthetically active radiation (PAR) drought, high and low temperatures, inappropriately high concentration of Zn^2+^, Cu^2+^ and Cd^2+^, air pollution, herbicides, mechanical damage, pathogen attack, etc.), however, it is also essential for normal cell growth and development (Jia et al., 2013). Also, in response to varied adverse environmental conditions, class III peroxidases from apoplasts might be a source of ROS, contributing to oxidative burst, alongside with NOX. These peroxidases could form ^•^OH within the presence of NADH, also as H_2_O_2_ (Swanson and Gilroy, 2010). ROS appearing within the apoplast may originate from other enzymes of the cell wall, for instance, oxalate oxidase also referred to as Germin, which releases H_2_O_2_ and CO_2_ from oxalic acid (Sharma et al., 2012).

### Role of ROS in Signal Transduction

Reactive oxygen species do not have an exclusively detrimental effect on the cell and its components. Namely, increasing attention is focused on the benefits of ROS for plants since ROS support cell proliferation, physiological processes, and viability and maintaining the basal level of ROS within the cell is specifically important. ROS, created by various enzymes in plants, perform fine-tuning of signal transduction process associated with plant growth and defense against biotic and abiotic stressors. Regulated production of low concentrations of ROS features a signal role. RBOH-dependent ROS are related to plant’s defense response to pathogens but also with plant growth. Namely, a temporary ROS increase within the apoplast is essential for leaf and root growth and differentiation (Suzuki et al., 2011; Morales and Munné-Bosch, 2016). Furthermore, peroxidases within the apoplast are involved in ROS signaling and accumulation in various cellular compartments, including chloroplasts, mitochondria, peroxisomes, and nucleus (Mittler, 2017).

**Figure 3:**
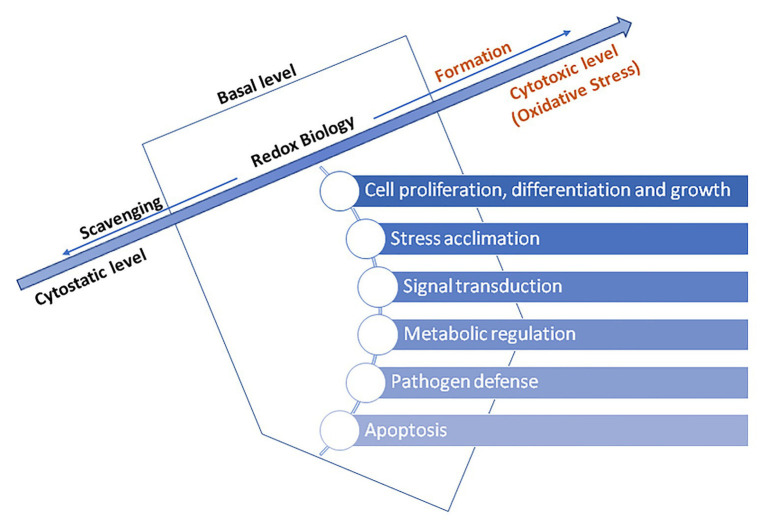
The basal level ROS-induced regulation of essential processes in a plant cells – adapted from: Mittler (2017; https://creativecommons.org/licenses/by-nc-nd/4.0/).

Therefore, ROS act as activators of signaling pathways for biological processes initiation. Signal translation mediated by redox reactions occurs primarily by oxidation and reduction of cysteine residues. Hence, for instance, H_2_O_2_ mediated oxidation of cysteine residues occur within the presence of nanomolar concentrations of H_2_O_2_. In contrast, H_2_O_2_ present in higher levels may irreversibly oxidize thiolate anions to sulfuric (SO_2_^−^) or sulfonic (SO_3_^−^) species, consequently promoting oxidative damage of biomolecules (Schieber and Chandel, 2014). For this reason, cells have enzymes which prevent the formation of intracellular H_2_O_2_, for instance, peroxiredoxins (PRX), glutathione peroxidase (GPx, EC 1.11.1.9), and ascorbate peroxidase (APX, EC 1.11.1.11; Exposito-Rodriguez et al., 2017; Mittler, 2017). Hence, a dominant concept in redox transmission is the balance between prooxidants on the one hand and antioxidants on the opposite. Counting on the oxidation degree, triggered programmed cell death and/or acclimatization of the plant and increased stress tolerance may occur (Jia et al., 2013; Noctor et al., 2018). Numerous authors emphasize the importance of maintaining the basal level of ROS above cytostatic and below cytotoxic, which allows redox reactions and essential processes regulation to happen (Truong and Carroll, 2013; Schieber and Chandel, 2014; Reczek and Chandel, 2015; Diebold and Chandel, 2016; Mittler, 2017). Too high or too low level of ROS impairs plant growth and development while sustaining an optimal level improves its progress, and therefore, responses to ROS are considered as dose-dependent. ROS and hormonal signaling are tightly intertwined where ROS acts as intrinsic growth and development signals activating many essential developmental processes, such as root hair growth, root elongation and gravitropism (thru auxin), stomatal closure (thru abscisic acid, ABA), lignin synthesis (thru jasmonic acid), leaf shape, trichome development, seed germination, etc. Beside phytohormones are involved in those adaptive responses of plants to environmental conditions, gibberellic acid (GA) is involved in process of apoptosis (Mhamdi et al., 2010; Gupta and Chakrabarty, 2013; Han et al., 2013; Talaat et al., 2019). Namely, GA-induced degradation of nuclear growth-repressing regulators, called DELLA proteins, is the key component of this mechanism. Additionally, it has been shown that triggering of programmed cell death by GA is tightly-related with ROS, predominantly H_2_O_2_, in aleurone cells of barley (Ishibashi et al., 2012). GA decreases the activity of catalase (CAT), ascorbate peroxidase (APX), and superoxide dismutase (SOD), leading to reduced scavenging ability for ROS, consequently resulting in peroxidative damage of membranes followed by release of hydrolytic enzymes (Jones and Smirnoff, 2005; Figure 3).

## Plant Antioxidant Defense System

Excessive oxidation and reduction of cell components are equally detrimental, so maintaining redox homeostasis is crucial (Foyer and Shigeoka, 2011). For this reason, plants are extremely rich in compounds with antioxidative activity. Although antioxidative protection is different from species to species, its presence is ubiquitous (Wachter et al., 2005; Pulido et al., 2009; Diaz Vivancos et al., 2010a,b).

**Figure 4:**
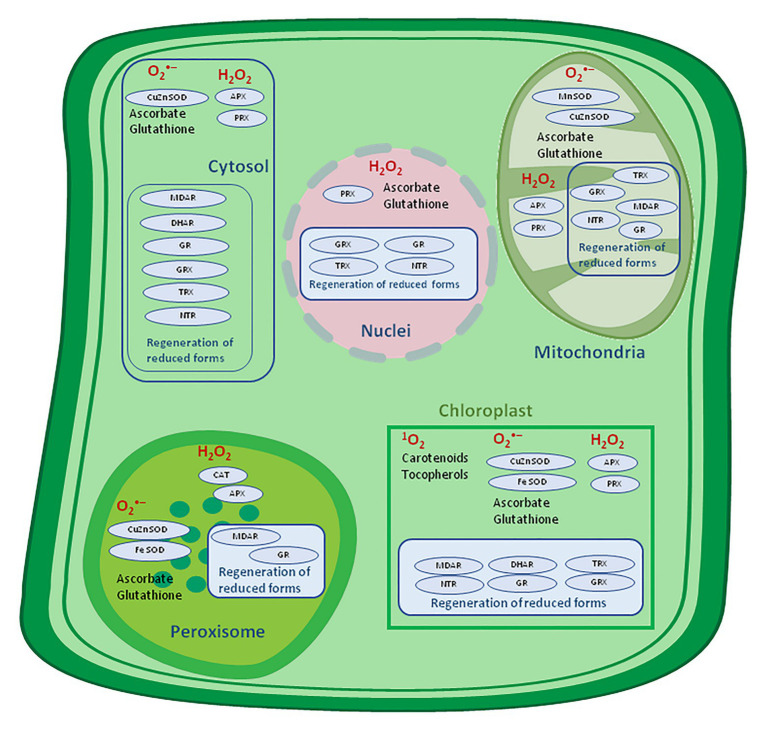
Antioxidative system location in a plant cell. APX, ascorbate peroxidase; CAT, catalase, DHAR, dehydroascorbate reductase; MDAR, monodehydroascorbate reductase; GR, glutathione reductase; GRX, glutaredoxin; SOD, superoxide dismutase; NTR, NADPH-thioredoxin reductase; PRX, peroxiredoxin; TRX, thioredoxin – adapted from: Noctor et al. (2018; http://creativecommons.org/licenses/by-nc-sa/4.0).

By definition, antioxidants represent molecules capable of inhibiting or quenching free radical reactions and delaying or preventing cell damage, and, in lower concentration than potential substrate which might be oxidized, significantly delay or hinder its oxidation (Nimse and Pal, 2015; Dumont and Rivoal, 2019). The foremost prominent low relative molecular mass antioxidants in plants are water-soluble ascorbate (Asc), glutathione, and phenols, and liposoluble tocopherols, tocotrienols, and carotenoids (Figure 4).

These molecules could self-react with ROS, but the removal efficiency is higher in enzyme-mediated reactions, like those catalyzed by APX (EC 1.11.1.11), ascorbate oxidase (AscO, EC 1.10.3.3), SOD, catalase (CAT, EC 1.11.1.6), and GPx (EC 1.11.1.9; Allen, 2009; Mhamdi et al., 2012; Noctor and Foyer, 2016; Noctor et al., 2018). Low relative molecular mass antioxidants remove ROS both indirectly and directly. Specifically, the indirect mechanism is chelation of transition metals, which prevents participation within the Haber-Weiss (Kehrer, 2000) or Fenton reaction, while the direct mechanism involves donating or receiving of electrons, scavenging radicals, and consequently preventing their reaction with biological molecules.

The antioxidant, which donates or receives electrons, is stabilized by π-electrons delocalization and resonance, and this is the case with Asc, phenolic compounds, and tocopherols. However, the advantage of scavengers over enzymatic antioxidants is their small size, which allows them to diffuse through cell membranes and localize near biological molecules which are potential targets of ROS (Kohen and Nyska, 2002). Additionally to those primary antioxidants, biomolecules, such as amino acids, sugars, pigments, also as secondary metabolites like flavonoids and terpenes, own antioxidant activity. Furthermore, secondary antioxidants are capable of regenerating oxidized primary antioxidant, as exemplified by Asc capable of regenerating oxidized α-tocopherol and α-tocopherol further regenerate β-carotene. Also, both created liposoluble radicals are often reduced by Asc, thereby exhibiting their antioxidant action within the membrane protection against lipid peroxidation. This is often an example of the synergistic action of AOS in preserving membrane integrity (Yachandra et al., 1996).

The most significant antioxidant in plant tissue, present at millimolar concentrations in chloroplasts, is Asc, followed by glutathione (GSH), which is present at 1,000 times lower concentration than Asc but is additionally vital. Specific enzyme systems (peroxidases) create the chance to rapidly react with H_2_O_2_, and their oxidized forms are regenerated by specific high-capacity reductases. In most cases, the entire amount of those antioxidants is greatly reduced (over 95%) within the cytosol, chloroplast, and mitochondria, with oxidized forms accumulating only in compartments with less efficient redox recycling mechanisms, like vacuoles and apoplasts (Noctor et al., 2018). Although these antioxidants scavenge ROS separately, they have long been thought to co-operate within the so-called water-water (Asada, 1999) and Asc-GSH cycle (Foyer and Halliwell, 1976) to metabolize H_2_O_2_ and keep sufficient excitatory energy under control within the chloroplasts (Foyer and Shigeoka, 2011). On the contrary, catalases play a serious role in H_2_O_2_ metabolism in peroxisomes (Mhamdi et al., 2012).

### Water-Water and Asc-GSH Cycle

**Figure 5:**
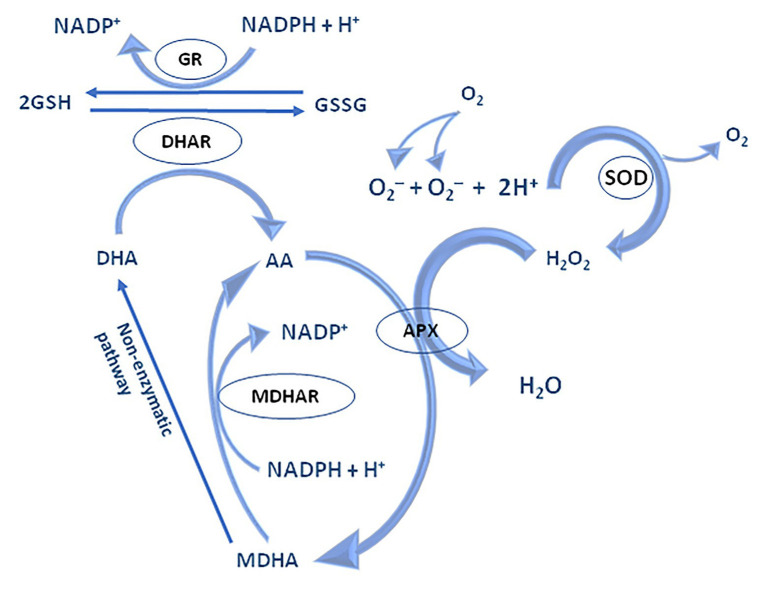
The mechanism of water-water and Asc-GSH cycle – adapted from: Noctor and Foyer (1998; https://creativecommons.org/publicdomain/mark/1.0/).

The water-water cycle begins by reducing the ground state of molecular oxygen to O_2_^•−^ on the acceptor side of PS I in Mehler’s reaction. Under physiological conditions, O_2_^•−^ is rapidly reduced to water by superoxide dismutase (SOD; Asada, 1999). However, O_2_^•−^ could even be non-enzymatically disproportionate to H_2_O_2_ and O_2_. The resulting H_2_O_2_ is detoxified by APX which in turns oxidizes two Asc molecules, its specific electron donor. Simultaneously, the formation of short-lived radical, monodehydroascorbate (MDA^•^), happen, which may be spontaneously converted to Asc and dehydroascorbate (DHA), and/or might be rapidly reduced to Asc by NAD(P)H-dependent monodehydroascorbate reductase (MDAR, EC 1.6.5.4; Foyer and Shigeoka, 2011). MDAR is found within the cytosol, chloroplasts, peroxisomes, mitochondria, and the plasma membrane (Noctor and Foyer, 1998). Unlike Asc, DHA lacks antioxidative capability and is converted back to Asc by the addition of two electrons from GSH by DHA reductases (DHAR, EC 1.8.5.1; Nimse and Pal, 2015). GSH-dependent DHAR activity is expressed in chloroplasts, mitochondria, and peroxisomes (Jimenez et al., 1997; Scheibe et al., 2005; Malik et al., 2011; Queval et al., 2011; Noctor and Foyer, 2016). GSH is regenerated from its oxidized state, glutathione disulphide (GSSG), by the action of glutathione reductase (GR, EC 1.8.1.7) using electrons from NAD(P)H, thus closing the regeneration cycle of Asc and GSH (Figure 5).

The Asc-GSH cycle is operating in various cellular compartments including the cytosol, mitochondria, chloroplast, and peroxisomes. Different isoforms of APX and SOD are localized within the stroma and thylakoid membrane, whereas chloroplast GR and DHAR are located within the stroma (Sharma et al., 2012). Despite the Asc-GSH cycle significance, newer evidence suggests overlapping of the role of peroxiredoxins (PRX) in maintaining a relevant level of H_2_O_2_ in chloroplasts.

Peroxiredoxins, which belong to the family of peroxidases, are important in ROS detoxification since they reduce H_2_O_2_ and organic peroxides and add cooperation with thioredoxin (TRX) and TRX-like proteins in chloroplasts (Dietz, 2011). PRXs utilize thiols to scale back H_2_O_2_. The Asc-GSH cycle has greater specificity for H_2_O_2_, and chloroplast APX has greater activity than PRX, however, PRXs are specific for lipid peroxides and/or RNS (Foyer and Noctor, 2016).

### Enzymatic Antioxidants

#### Superoxide Dismutase

Superoxide dismutase plays a serious role in oxidative stress by catalyzing the rapid dismutation of O_2_^•−^ and thus reducing the danger of ^•^OH formation *via* metal-catalyzed reactions. SOD-catalyzed dismutation is 10,000 times faster than spontaneous reactions. The enzyme is present in all aerobic cells and subcellular compartments sensitive to oxidative stress (Bowler et al., 1992). There are three types of SOD metalloenzymes in plants counting on metal cofactor present within the active center. The foremost abundant isoenzyme is Cu/Zn-SOD found in chloroplast stroma, cytosol, peroxisomes, and apoplast. Mn-SOD is expressed in mitochondria and peroxisomes but has also been detected in both apoplast and cell wall, while Fe-SOD is present to a lesser extent. However, this isoenzyme is restricted for the chloroplast stroma of certain plant species (Mittler et al., 2004).

These isoenzymes are differentiating in their sensitivity to H_2_O_2_ and KCN (Bannister et al., 1987). Genes coding SOD are sensitive to environmental stressors, and increased activity is usually correlated with increased plant tolerance for environmental stress (Sharma et al., 2012). SOD acts as the first level of protection against ROS, transforming O_2_^•−^ into H_2_O_2_, and APX, GPx, and CAT further detoxify the resulting H_2_O_2_ (Apel and Hirt, 2004). The *knock-down* mutants of chloroplast Cu/Zn-SOD show suppressed growth, reduced size of chloroplasts, and impaired photosynthetic activity (Rizhsky et al., 2003).

#### Catalase and Peroxidase

The intracellular level of H_2_O_2_ is regulated by several enzymes, the foremost important of which are catalases (CATs) and peroxidases participating within the fine regulation of ROS concentration through the cell (Baby and Jini, 2010). Considering cellular compartmentalization, CATs might be found in large quantities in peroxisomes, whereas this enzyme has not been found in chloroplasts. It captures the H_2_O_2_ created in peroxisomes during the process of photorespiration and β-oxidation of fatty acids. The catalases contain four heme subunits with Fe^2+^ ions undergoing oxidation and catalyze the dissociation of two H_2_O_2_ molecules into water and oxygen (Arora et al., 2002).

Catalases are very efficient in H_2_O_2_ removal with a unique ability to convert two H_2_O_2_ molecules into water and molecular oxygen with no need for reduction equivalent. Precisely, this happens *via* oxidation of Fe^2+^ ion in heme, after which Fe^2+^ is reduced by reaction with H_2_O_2_. The *Km* value for CAT is within the millimolar range, which may be a far higher concentration of H_2_O_2_ within the cell than physiological, implying its role predominantly under stress conditions (Černý et al., 2018). On the other hand, CATs express low activity against organic peroxides. Comparing with peroxidases, they have higher *Km* value for H_2_O_2_ hence peroxidases could remove H_2_O_2_ albeit present in low concentrations (Sharma et al., 2012). On the other hand, peroxidases-driven reactions are using low relative molecular mass antioxidants, GSH and Asc as electron donors, so removal of H_2_O_2_ using this pathway may be a very energy-consuming reaction for cell since it utilizes important molecules from the cell environment: two GSH molecules are consumed for removal of one H_2_O_2_ molecule (Kohen and Nyska, 2002). Regarding the aminoalkanoic acid sequence, peroxidases are divided into three classes (Welinder, 1992). The APX is assessed as a first-class and differs from the class III peroxidase-like well-known horseradish peroxidase (HRP). The importance of APX within a plant cell is indicated by the very fact that APX isoenzymes are distributed in as many as five cell compartments: stroma (sAPX) and thylakoids (tAPX) in chloroplasts, microbodies (including glyoxysomes and peroxisomes; mAPX), cytosol (cAPX), and mitochondria (mitAPX, as a membrane-bound form; Chen and Asada, 1987; Jimenez et al., 1997; De Leonardis et al., 2000). Mutants with tAPX deficiency are considered lethal while plants with overexpressed tAPX are tolerant of methyl viologen (paraquat) induced stress (Yabuta et al., 2002). Also, overexpression of cytosolic APX in chloroplasts led to the rise of plant’s tolerance to drought and high salinity (Badawi et al., 2004), whereas *knock-out* mutants have compromised chloroplast scavenging system and increased H_2_O_2_ accumulation in leaves (Davletova et al., 2005). However, APX isoenzymes aren’t so effective in reducing lipid hydroperoxides. A salient feature of APX, especially chloroplast, is its sensitivity to oxidative inactivation within the absence of Asc. At low concentrations of Asc (less than 20 mmol/L), chloroplast APX activity is rapidly decreased in the presence of H_2_O_2_, with an inactivation half-life of the 30 s. In contrast, cytosolic and peroxisomal isoforms lose their activity after quite 1 h (Miyake and Asada, 1996). Depletion of chloroplast Asc and inactivation of chloroplast APX are considered for limitations of photosynthetic efficiency under stress conditions and thus potential targets for improvement (Ishikawa and Shigeoka, 2008). The sole H_2_O_2_-scavenging enzyme within the apoplastic and vacuolar space of all plant organs is class III peroxidase. They catalyze the oxidation of different substrates with H_2_O_2_ acting as an electron acceptor, consequently generating radicals (mainly phenolic), and such reaction is described as Peroxidase/Phenolics/Ascorbate (PPA) system (Takahama, 2004). Induced in response to various environmental stresses, this family of isoenzymes has an imperative role in cross-talk between primary and secondary antioxidants (Veljović-Jovanović et al., 2018).

Glutathione peroxidases, which even have strong activity against H_2_O_2_, could use both GSH and TRX as reducing substrates and will eliminate lipid peroxides additionally to H_2_O_2_ (Herbette et al., 2002). Besides their role in H_2_O_2_ neutralization, CAT and GPx together with SOD enzyme show a synergistic effect in O_2_^•−^ elimination.

### Non-enzymatic Antioxidants

#### Ascorbic Acid

Ascorbate is taken into account a potent antioxidant thanks to its ability to donate electrons in an exceedingly wide selection of enzymatic and non-enzymatic reactions. It is especially present within the leaves and in higher concentration compared to GSH (Das and Roychoudhury, 2014). Under physiological conditions, within the sort of monoanionic, Asc mainly exists in its reduced form (90%) in chloroplasts, with the remainder within the sort of DHA which lacks antioxidative activity (Yachandra et al., 1996; Noctor and Foyer, 1998). Ascorbate could donate two electrons, whereby donation of one is followed by the assembly of semidehydroascorbate or ascorbate, and donation of the second electron is related to DHA production. It might be regenerated by DHAR utilizing two GSH molecules.

Ascorbate occurs in all subcellular compartments including the cell wall except for vacuoles where is present in low concentrations (Das and Roychoudhury, 2014). Nevertheless, the bulk of Asc is found within the cytoplasm, but unlike other soluble antioxidants, a substantial portion is exported to the apoplast where it is present at millimolar concentrations and is taken into account to be the primary line of defense against potentially harmful external prooxidants. Chloroplast’s Asc is also a cofactor for violaxanthin de-epoxidase where it participates within the production of xanthophylls which are directly involved in quenching of excessive excitatory energy on PS II (Davey et al., 2000).

Ascorbate, as quantitatively dominant antioxidant in plant cells, is found altogether subcellular compartments including the apoplast with a mean concentration of 2–25 mmol/L or more within the chloroplast stroma (Foyer and Lelandais, 1996). Intracellular concentrations are up to millimolar range (i.e., 20 mmol/L within the cytosol and 20–300 mmol/L in chloroplast stroma; Larson, 1988). Asc can directly capture ^•^OH, O_2_^•−^, and ^1^O_2_ and also to reduce H_2_O_2_ to water *via* an ascorbate peroxidase reaction (Noctor and Foyer, 1998). However, Asc could acts as prooxidant reducing Fe, Cu, and Mn ions and consequently providing a chance to re-engage in one among the redox reactions.

#### Glutathione

The tripeptide, γ-glutamyl-cysteinyl glycine, the foremost abundant low relative molecular mass thiol within the cell, has been found in large quantities in every cell compartment: cytosol, chloroplast, endoplasmic reticulum, vacuoles, and mitochondria. It is not only specific to plant cells but also plays a really important role as a redox buffer amid Asc (Liu and Li, 2019).

The reduced form of glutathione, GSH, may be a major sulfur depo form and plays important roles in various biological processes, including cellular growth, development, regulation of sulfur transport, signal transduction, protein and nucleic acid synthesis, phytochelatin synthesis for metal chelation, xenobiotic detoxification, and expression of genes liable for stress (Bartoli et al., 2017). Besides, GSH is synthesized both in chloroplasts and within the cytosol. It can chelate Cu^2+^ ions and cease them from participating in the Haber-Weiss reaction (Zlobin et al., 2017).

Together with its oxidized form, GSSG, reduced glutathione maintains redox balance within the cell. The cysteine residue in the molecule center is liable for the high reduction potential of GSH. As low relative molecular mass antioxidants, GSH could scavenge H_2_O_2_, or react non-enzymatically with ^1^O_2_, O_2_^•−^, and ^•^OH (Krasnovsky, 1998). However, the main role of GSH as an antioxidant is its ability to regenerate another potent hydrophilic antioxidant, ascorbic acid, precisely through the Asc-GSH cycle. GSH helps to recycle oxidized Asc to the reduced state employing DHAR. GSH also can reduce DHA non-enzymatically at pH > 7 and GSH concentrations >1 mmol/l (Sharma et al., 2012).

#### Carotenoids

Carotenoids, such as lycopene, β-carotene, xanthophyll, lutein, and zeaxanthin, are lipophilic antioxidants capable of detoxifying various ROS and most effectively capture the lipid peroxyl radical (LOO^•^), thus providing membrane protection. Carotenoids react with LOO^•^ and form lipid hydroperoxide (LOOH) and a carotenoid radical which will be regenerated by tocopherol, and both tocopherol and carotenoid radicals might be reduced by Asc subsequently (Yachandra et al., 1996).

Present in plants, they may capture ^3^Chl, ^1^O_2_, also as excited chlorophyll (Chl^∗^) to guard the photosynthetic apparatus. Hence, β-carotene captures ^1^O_2_ with greater efficiency compared to α-tocopherol (Kehrer, 2000; Sharma et al., 2012). The conjugated double bond system owned by carotenoids provides easy absorption of energy from the excited molecule and dissipation of excess within the sort of heat. For instance, zeaxanthin is involved within the non-photochemical quenching of excess excitatory energy at PS II (Asada, 1999).

#### Tocopherols and Tocotrienols

Tocopherols and tocotrienols are essential components of the cell membrane where they express both antioxidant and non-antioxidant functions. There are four tocopherol and tocotrienol isomers (α, β, γ, and δ). Tocopherols are a gaggle of lipophilic antioxidants and are synthesized by photosynthetic organisms and present in green, photosynthetically active parts of the plant only. The antioxidant activity of tocopherol is predicated on the electron donor properties of the chromanol ring.

These antioxidants protect lipids and other membrane components by physically trapping and chemically reacting with ^1^O_2_ in chloroplasts, preserving the structure and performance of PS II. The method of ^1^O_2_ capture is extremely efficient and it has been estimated that 1 α-tocopherol molecule can neutralize up to 220 molecules of ^1^O_2_ *in vitro* before its degradation (Sharma et al., 2012). However, their rate is two times lower compared with β-carotene and they are not effective in capturing ^•^OH and alkoxy radicals (RO^•^) *in vivo* (Kohen and Nyska, 2002; Blokhina et al., 2003). Regeneration of oxidized tocopherol might be achieved *via* Asc, GSH, or ubiquinone.

The relative antioxidant activity of the isomers *in vivo* corresponds to the subsequent order α > β > γ > δ due to the methylation pattern and the number of methyl groups added to the polar head of the phenolic ring. Also, α-tocopherol, with its three methyl substituents, has the very best antioxidant activity. However, α-tocotrienol has been shown to possess better antioxidant activity than α-tocopherol within the membrane environment. The chloroplast membrane contains predominantly α isomer of tocopherol; therefore, they are well protected from photooxidative damage (Smirnoff, 2000). Furthermore, α-tocopherol is the main form within the leaves, while γ-tocopherol is within the seed.

Vitamin E (collective term for tocopherols and tocotrienols) has the potential for regenerating lipid peroxyl, alkyl, and alkoxy radicals formed during the polyunsaturated fatty acids oxidation whereby directly prevent a sequence propagation during auto-oxidation of the lipid layer. By donating hydrogen atoms to the radical, vitamin E becomes tocopherol radical which is resonantly stabilized and not sufficiently reactive for independent initiation of membrane peroxidation, which is additionally a basic criterion for good antioxidants (Kohen and Nyska, 2002). Also, tocopherol present in high concentrations acts as prooxidant alongside transition metal ions and lipid peroxides.

#### Phenolic Compounds

Phenols are a multifarious group of secondary metabolites (flavonoids, tannins, hydroxycinnamate esters, lignin, etc.) present in plant tissue (Sakakibara et al., 2003). The antioxidant capacity of phenols is related to their structure (aromatic ring with –OH or –OCH_3_ substituents) very suitable for trapping free radicals. They have a robust capacity to donate an electron or hydrogen atom also because the ability to rapidly stabilize formed phenol radical and have shown greater activity compared to Asc and α-tocopherol in *in vitro* system. Phenols containing *o*-dihydroxy groups within their structure can complex metal ions and prevent the formation of ROS in the Haber-Weiss reaction (Fenton, 1984; Rice-Evans et al., 1996). Furthermore, they will directly capture ^1^O_2_ and inhibit lipid peroxidation by trapping lipid alkoxy radicals (Sharma et al., 2012). Another mechanism associated with antioxidant properties of phenols is the ability to modify the kinetics of peroxidation by altering the lipid package and reducing membrane fluidity. These changes can limit the diffusion of free radicals and reduce the peroxidation reaction. It has also been shown that phenolic compounds could be involved within the H_2_O_2_ capture cascade (Takahama and Oniki, 1992). Increased accumulation of phenolic compounds under biotic also as abiotic stress has been demonstrated and certain anthocyanins and flavanols have up to fourfold higher antioxidative activity than Asc (Rice-Evans et al., 1996).

The cooperation between Asc and phenols has been shown within the hydrogen-peroxide-peroxidase system which takes place in vacuole where H_2_O_2_ diffuses and may be reduced by peroxidases, and phenols are used because of the primary electron donors. Both Asc and MDA^•^ radicals can reduce the phenoxy radical. If Asc regeneration takes place within the cytosol and Asc is delivered back to the vacuole, the peroxidase/phenols/Asc system can operate within the vacuole and capture H_2_O_2_. This mechanism is restricted to plant tissue and enhances plant tolerance during oxidative stress (Krasnovsky, 1998). The MDAR enzyme has also been shown to be capable of reducing the phenoxy radical, like quercetin radical, to phenol (Sakihama et al., 2000).

## Conclusion and Outlooks

Accordingly, available data indicate that ROS detoxification pathways are not present to the extent they might remove all ROS from the cellular environment, but that there is a level of coordination between the processes which generate ROS and those which remove them, therefore, maintaining the optimal amount of ROS within the cellular environment.

A really popular trend for testing AOSs is the use of genetically transformed plants, with overexpressed or removed a selected component of AOS, as well as the application of artificial environmental conditions to cause oxidative stress. Extensive literature indicates that enhancing the expression of certain enzymes likes SOD, GR, and DHAR, utilizing gene-splicing, can improve plant tolerance to abiotic stress. Certainly, the enhancement of chloroplast antioxidative protection has been proven to be one among the foremost effective pathways for shielding plant cells from abiotic stress.

In addition to the fact that antioxidants are essential for plant’s subsistence, they may benefit humans and well. This claim has been supported by plenty of antioxidant formulations offered and available to us in markets. The main constituents of those formulations are principally plant’s extracts containing biologically active compounds well-known for some favorable effect. For instance, it has been investigated that natural compounds could help in the prevention of neurodegenerative diseases for instance. Also, the fact that many of those antioxidants cannot be synthesized within human cells due to lack of enzymes in the first place, qualify them as essential nutrients for our population.

Extensive research is being conducted to investigate natural compounds which may curb or alleviate oxidative stress and thereupon empower the immune system and nowadays, we have a growing number of plant-based nutrition supporters. The last decade is supported by investigations of potentially beneficial mild prooxidant effects. Namely, moderate-dose exposure to noxious agents or factors induces an adaptive response of cells termed as *hormesis*. Overall, although six decades-long, this multiplex field of research is still dynamic and subject to evolve due to acquiring deeper insights and new knowledge of this intricate network of molecules and their reactions. Although particular antioxidant compounds express extraordinary antioxidant capacity *in vitro*, more challengeable and complex *in vivo* studies, which will perfectly simulate an intracellular environment with the presence of an orchestrated network of pro‐ and antioxidants should be conducted.

## Author Contributions

JD and VJ: conceptualization, investigation, resources, and writing – original draft preparation and visualization. VJ, EN, MN, and KK: validation. MN and VJ: formal analysis, writing – review, and editing. KK: supervision and funding acquisition. EN: project administration. All authors have read and agreed to the published version of the manuscript.

### Conflict of Interest

The authors declare that the research was conducted in the absence of any commercial or financial relationships that could be construed as a potential conflict of interest.
